# Image-Guided Localization Techniques for Surgical Excision of Non-Palpable Breast Lesions: An Overview of Current Literature and Our Experience with Preoperative Skin Tattoo

**DOI:** 10.3390/jpm11020099

**Published:** 2021-02-04

**Authors:** Gianluca Franceschini, Elena Jane Mason, Cristina Grippo, Sabatino D’Archi, Anna D’Angelo, Lorenzo Scardina, Alejandro Martin Sanchez, Marco Conti, Charlotte Trombadori, Daniela Andreina Terribile, Alba Di Leone, Beatrice Carnassale, Paolo Belli, Riccardo Manfredi, Riccardo Masetti

**Affiliations:** 1 Multidisciplinary Breast Centre, Dipartimento Scienze della Salute della Donna e del Bambino e di Sanità Pubblica, Fondazione Policlinico Universitario A. Gemelli IRCCS, 00168 Rome, Italy; gianlucafranceschini70@gmail.com (G.F.); sabatinodarchi@gmail.com (S.D.); lorenzoscardina@libero.it (L.S.); martin.sanchez@hotmail.it (A.M.S.); daniterribile@gmail.com (D.A.T.); albadileone@gmail.com (A.D.L.); carnassale.beatrice@gmail.com (B.C.); riccardo.masetti@policlinicogemelli.it (R.M.); 2 Dipartimento di Scienze Mediche e Chirurgiche, Università Cattolica del Sacro Cuore, 00168 Rome, Italy; 3 Dipartimento di Diagnostica per Immagini, Radiologia Terapeutica ed Interventistica, Azienda Ospedaliera Santa Maria Terni, 05100 Terni, Italy; cris.grippo@gmail.com; 4 Dipartimento di Diagnostica per Immagini, Radioterapia Oncologica ed Ematologia, Fondazione Policlinico Universitario Agostino Gemelli IRCCS, 00168 Rome, Italy; anna.dangelo05@gmail.com (A.D.); conti.marco87@gmail.com (M.C.); charlotte.trombadori@gmail.com (C.T.); paolo.belli@policlinicogemelli.it (P.B.); riccardo.manfredi@policlinicogemelli.it (R.M.)

**Keywords:** breast cancer, breast-conserving surgery, non-palpable breast lesions, image-guided localization, preoperative breast localization, breast ultrasound

## Abstract

Breast conserving surgery has become the standard of care and is more commonly performed than mastectomy for early stage breast cancer, with recent studies showing equivalent survival and lower morbidity. Accurate preoperative lesion localization is mandatory to obtain adequate oncological and cosmetic results. Image guidance assures the precision requested for this purpose. This review provides a summary of all techniques currently available, ranging from the classic wire positioning to the newer magnetic seed localization. We describe the procedures and equipment necessary for each method, outlining the advantages and disadvantages, with a focus on the cost-effective preoperative skin tattoo technique performed at our centre. Breast surgeons and radiologists have to consider ongoing technological developments in order to assess the best localization method for each individual patient and clinical setting.

## 1. Introduction

Breast cancer (BC) is the most commonly diagnosed cancer and the leading cause of cancer-related death among women [1]. A successful BC treatment is based on a multidisciplinary use of surgery, chemotherapy and radiation therapy, with surgery as the central component of treatment for early-stage breast cancer [2,3]. Breast-conserving surgery (BCS) followed by adjuvant radiotherapy, known as breast conservation therapy (BCT), has become the alternative treatment to mastectomy for early stage breast cancer because of equivalent survival and lower morbidity [4,5,6].

Local recurrence after BCS is strongly correlated to the surgical margin status, as demonstrated by a large number of follow-up studies [7,8,9,10,11]. The main goal of BCS is to fully remove the tumor with clear margins, while avoiding resection of healthy breast tissue in order to achieve better cosmetic results. Image-guided preoperative localization is mandatory for guiding surgery of non-palpable lesions or surgically relevant extension of palpable lesions to improve both oncological and cosmetic outcomes [12,13]. Over the last decade, methods for preoperative localization of breast lesions for BCS have evolved rapidly due to innovative techniques and discovery of novel agents. However, cooperation and communication between breast surgeons and radiologists still play a crucial role.

Different image guided localization techniques are variably used in different institutions depending on personal choices, skills and available technologies. As a general rule, the method chosen should be the most precise to localize the lesion or marker left after biopsy, thus improving free margin rates and decreasing operative time, and possibly cause little to no discomfort to the patient. Preoperative breast lesions localization techniques currently available are wire localization, carbon marking, radio-guided occult lesion localization (ROLL), radioactive seed localization (RSL), magnetic seed localization and non-radioactive radar localization, intraoperative ultrasound and preoperative skin tattoo localization (Table 1). In this article, we provide an overview of current literature of all commercially available techniques. The aim of this review is to educate practicing radiologists and breast surgeons so they can knowingly select new techniques to improve patient care.

## 2. Wire Guided Localization

**Figure 1:**
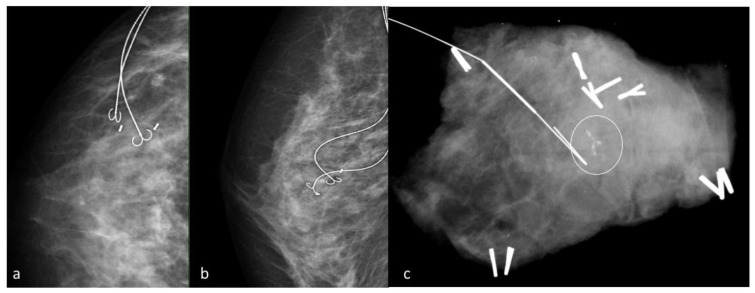
Wire-guided localization. Craniocaudal (**a**) and mediolateral (**b**) oblique mammograms taken after hookwires insertion show optimal wires positioning, with the wires at the biopsy markers site. A specimen radiograph (**c**) contains the hookwire and the residual calcifications (circle).

Wire localization (WL) was introduced in the 1970s and for many years has served as the only method for preoperative breast localization [36]. Initially, mammography was the only imaging modality used to guide wire placement. Currently, wire localization can be performed under different kinds of image-guidance (mammographic, sonographic and magnetic resonance imaging). WL is the most commonly used method for non-palpable breast lesions, with clear margins reported in a range of 70.8%–87.4% of cases [15]. Different types of wires are available, ranging in length (from 3 to 15 cm), shape (hook, barb or pigtail), materials and numbers of thickened segments [12,13,15,36,37]. Wires are preloaded in a 16–21 G needle introducer: when the tip is just beyond the target, the hook is deployed by fixing the needle firmly with one hand and gently advancing the wire with the other. The needle is then removed over the wire and the thread extending from the tip of the hookwire is secured on the skin surface. Routinely, post-procedural CC and ML mammograms were obtained to confirm accurate placement (Figure 1). The depth of the wire tip from the skin surface is also recorded. In case of extensive disease wires can be placed in multiple numbers, allowing targeted localization in a procedure known as “bracketing wire localization” [38]. WL remains the most widely adopted approach due to the long-term data supporting its effectiveness [39], although success is strongly dependent upon the surgeon’s mental reconstruction of the images, perceived intraoperative position of the lesion and wire trajectory [40]. Approximately 2.5% of wire localizations are unsuccessful; factors associated with an increased risk of unsuccessful localization are multiple lesions, small lesions, lesions containing extensive microcalcifications and small surgical specimens [14]. Established advantages of WL are the widespread availability and the moderate price, with one study estimating the cost of a needle at $22.50 [41].

Moreover, wires emit no ionizing radiation and can be stored safely within the imaging department. This approach also allows localizations of breast lesions under different kinds of image guidance (US, mammography/tomosynthesis or MRI). Although WL is highly effective, it still yields several disadvantages. The procedure is in itself unpleasant and causes patient discomfort; vasovagal reactions are reported in up to 7–10% of patients, although less frequent for US than for mammography guided procedures [12]. Wire migration within the breast, and more infrequently outside the breast, has also been reported [42,43]. The hookwire can be transected during the surgery, with pieces being retained in the breast post-operatively [44,45]. Finally, this localization approach requires adequate coordination between trained breast radiologists and surgeons because the wire placement has to occur on the day of surgery to avoid displacement. This limitation can lead to inconvenience and delay in the operating room or suboptimal localization. Moreover, wire localization could limit the surgical approach and cause a potential worse cosmetic outcome; the placement route of the wire, chosen by the radiologist, often dictates incision choice for the surgeon who then has to follow the wire’s course during dissection.

## 3. Carbon Marking

Carbon marking (CM) is an alternative method for non-palpable breast lesion localization first reported by Svane in 1983, consisting of an injection of sterile charcoal powder diluted with saline solution in close proximity to the lesion [46]. The injection can be performed under either sonographic or mammographic guidance, depending on how the target lesion has been biopsied [17]. A dark trail is created from the lesion to the skin, leaving a visible track that guides the surgeon during the operation. As the carbon track is immobile in breast tissue, it cannot dislodge. In contrast, hookwires can migrate when the patient changes position or when traction is applied during surgery. The main advantages of CM are logistics, patient comfort and cost. As CM and biopsies could be concurrent, the patient may be spared an extra invasive procedure. Moreover, surgery may be planned up to 1 month after the carbon injection, making operative planning easier for surgeons and sparing radiologists the pressure to place hookwires immediately before or during an operating session. The success rate using carbon marking is very high, with failure to remove targeted lesion occurring in about 1 in every 100 procedures [16]. However, there are cases in which CM presents technical difficulties. If the lesion is close to the chest wall, particularly in a large breast, or for extensive or multifocal lesions, long and several carbon tracks will be difficult for the surgeon to follow and a hookwire may be preferable. For extensive or multifocal lesions several carbon tracks are difficult to follow, and WL may be preferable [46]. The disadvantages are that the carbon tracks resist slicing, thus the carbon can distort or obscure the lesion. To avoid this, the carbon should be injected only as far as the edge of the lesion. Another possible, although uncommon, complication of CM is the incomplete surgical removal of the injected charcoal, which can cause a late-onset granuloma that may mimic malignant lesions in postoperative controls [47,48]. In terms of missed lesions and clear margin rates, CL shows similar results as WG: the proportion of cases with close or involved margins ranges between 15% (for invasive cancer) and 39% (in situonly lesions) [17,18].

## 4. Radio-Guided Occult Lesion Localization

**Figure 2:**
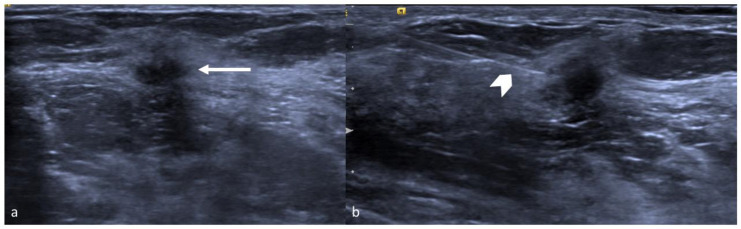
Radio-guided occult lesion localization (ROLL) technique: (**a**) invasive ductal cancer (arrow) in the left upper outer quadrant in a 77-year-old woman. (**b**) Intratumoral injection (arrow head) of a small amount (0.2–0.3 mL) of human serum albumin marked with nuclear radiotracer technetium 99 in order to perform radio-guided occult lesion localization.

Radio-guided occult lesion localization (ROLL) involves intratumoral injection of a small amount (0.2–0.3 mL) of human serum albumin marked with nuclear radiotracer technetium 99 [49] (Figure 2). This localization technique can be performed either by ultrasonography, stereotactic mammography or MRI.

The radiation dose is of about 7–10 MBq, equivalent to 1–2% of the dose used for a whole-body bone scintigraphy [50]. A handheld gamma ray detection probe is used by the surgeon to locate the lesion, guide the removal and verify the removed specimen and the surgical bed. To allow an adequate detection, surgery has to be performed no later than 24 h after the injection of the radiotracer. ROLL has gained popularity on account of several advantages associated with a reduced excision volume, more accurate centricity of a lesion within the surgical specimen, better cosmetic results and a higher percentage of tumor-free margins, around 92% of cases [20,21]. There are no serious complications related to ROLL, even though experience in the injection is needed to avoid failure of lesion identification, described only in 1–5% of the cases [19]. ROLL can be performed together with sentinel lymph node identification in the same surgical session, in a procedure known as sentinel node and occult lesion localization (SNOLL), that involves the injection of an additional radiotracer (carried by micromolecules instead of macromolecules used for ROLL) [51,52]. 

## 5. Radioactive Seed Localization

Radioactive seed localization (RSL) using Iodine-125 seeds has been proposed in 1999 by Dauway as an attractive alternative to both WL and ROLL. This technique involves targeted placement of a seed, commonly used for brachytherapy, composed of titanium labeled 0.075–0.3 mCi of Iodine-125. Each seed has a half-life (T 1/2) of 59 days and a radioactivity of about 20–30 MBq, a dose equivalent to 3–5% of that used for a whole-body bone scintigraphy [53]. Radioactive seeds can be positioned under different image guidance, ultrasonography, mammography/tomosynthesis or MRI. An 18G needle preloaded or manually loaded with the seed was used, and the tip was occluded by bone wax. Once the needle advanced to the desired location, the seed was deployed through the bone wax by advancing the stilette. At the end of the procedure, regardless of the guidance method, the patient was assessed for radioactivity with a Geiger counter and post-procedural mammograms with two orthogonal images reconfirm proper seed positioning [54]. During surgery a gamma probe set for I-125 guides the surgeon. The different energy peak of technentium-99 and iodine-125 allows one to differentiate the isotope used for sentinel node biopsy. Radioactive seed localization could potentially be performed weeks before the scheduled surgery because of the long half-life (59 days) of I 125; however, according to Nuclear Regulatory Commission guidelines the procedure should be carried out no more than 7 days before surgery in order to minimize radiation exposure [55]. In fact, one of the potential drawbacks of RSL is the presence of radioactivity. Although the activity levels of the seeds are low and considered safe for human exposure, patients are advised to avoid interactions with children and pregnant women to mitigate any potential risk. Moreover, a strict local protocol for quality assurance must be followed in order to guarantee that all implanted seeds are actually removed and recovered by the local Nuclear Medicine Department. An undeniable benefit of both ROLL and RSL is that the surgeon is no longer impeded by the guidewire when planning breast incision and can use the feedback from the gamma probe to reorientate the surgical approach in real time.

Given oncoplastic breast techniques, this allows greater choice of cosmetically sensitive approaches, such as periareolar, lateral or inframammary fold incisions [56]. Current literature comparing RSL and WL margin status achievements shows variable results, with some studies favoring RSL and more recent studies, including three randomized control trials, suggesting no difference between the two methods [40,57,58]. Due to the real-time intraoperative monitoring of the detected gamma counts from the seed, RSL allows an accurate lesion localization with lower incidence of positive margins and decreased need for repeat surgery than with wire localization. The success rate using RSL is very high: target lesion is effectively removed in nearly 100% of cases and clear margin rates range from 73.5% to 96.7% [22,23].

## 6. Magnetic Seed Localization

**Figure 3:**
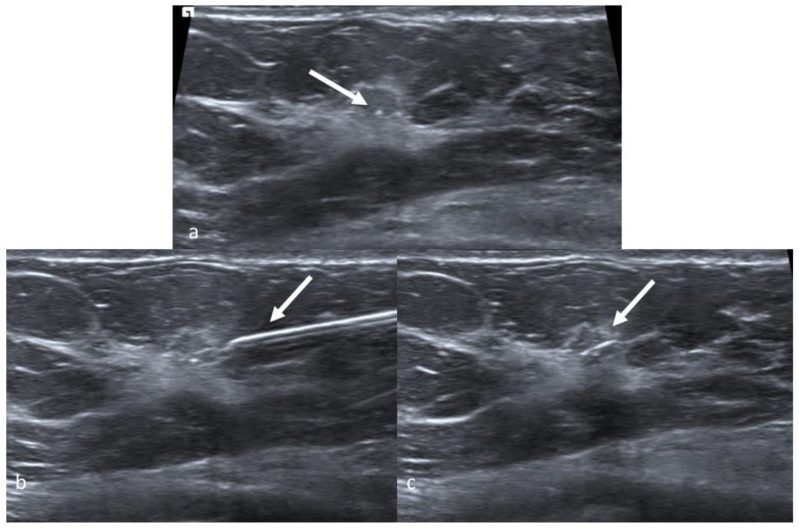
Magseed positioning in a 49-year-old woman with ductal carcinoma in situ. Ultrasound images of the right upper outer quadrant. Biopsy marker is visible in the lesion (**a**, arrow). Magseed magnetic marker is placed under ultrasound guidance (**b**, arrow shows the needle). Magseed^®^ marker is clearly seen in the lesion (**c**, arrow).

Magnetic seed is a novel localization technique approved by the FDA in 2016 [24]. This technique shares many similarities with RSL, because it consists in seed placement under sonographic or tomosynthesis guidance, however it does not involve radioactivity. First introduced by Sentimag (Magseed^®^, London, UK), magnetic seeds are cylindrical markers, measuring approximately 5 mm × 1 mm, made of paramagnetic steel and iron oxide. They can be deployed by an 18 G preloaded needle of different length according to different breast sizes (Figure 3). Following insertion, mammograms in double projection are acquired to confirm correct positioning of the seed. The Sentimag probe employed in the operating room generates an alternating magnetic field that temporarily magnetizes the Magseed, and subsequently measures its magnetic field. The surgical technique is therefore similar to that adopted after ROLL or RSL, involving a live numerical feedback that guides surgical direction and reveals the remaining distance from lesion. A final assessment is conducted by probing the specimen and the surgical cavity, and potentially verified with specimen X-ray confirming excision of seed. While sharing with ROLL and RSL the important benefit of granting maximum liberty in the choice of incision, this technique has the further benefit of avoiding exposure to radiation. It also eases coordination between Radiology and Surgery Departments, because seed placement, initially approved for up to 30 days prior to surgery, has now been extended in Europe and USA for long-term implantation [25]. However, while this seed could be potentially implantable during biopsies and even before neoadjuvant treatment, one major drawback is that it interferes with MR imaging by creating artifacts as wide as 4 cm [12]. Another challenge with this technique is that during surgery all ferromagnetic instruments will interfere with the signal. A dedicated set of non-ferromagnetic surgical instruments is therefore always necessary, and weighs on cost-effectiveness [13]. Studies on the efficacy of this technique in terms of successful excision, clear margins and optimal volume of resection are few and include relatively small populations of patients, however preliminary data is encouraging, with a successful placement rate of 94.42%, a successful localization rate of 99.86% and a percentage of clear margins of 88.75% [24,26,27,59].

## 7. Radiofrequency Identification Tags

Radio frequency reflector (RFR) is a non-ionizing electromagnetic wave tagging device for localizing non-palpable breast lesions approved in the United States by FDA in 2014 [28]. The identification tag, as any biopsy clip marker, can be placed by radiologists under mammographic, tomosynthesis or ultrasound guidance. The injection can take place up to 30 days preoperatively. During surgery, the surgeon activates the reflector with the hand piece and follows the signal to guide the excision. The audible and numerical signals change with increasing proximity to the lesion. Once the tissue is removed, the reader console can be used to confirm that all tags have been removed from the tissue cavity. RFRs differ in size and shape from vendor to vendor. One of the first available RFR is SAVI SCOUT (Cianna Medical, Aliso Viejo, CA, USA) and another more recent device is the LOCalizer (Faxitron, Tucson, AZ, USA). The SAVI SCOUT reflector has been rated as MR conditional and be considered safe to image in a static magnetic field of 3 Tesla or less and a maximum spatial gradient magnetic field of 3000 G or less [29]. Whereas metallic interference from nearby surgical instruments can interfere with detection of magnetic seeds, metal does not interfere with detection of radiofrequency signals during surgery [59]. Radiofrequency identification tag is an effective technique: data from the literature report success rates of 97–100% and clear margin rates ranging between 85% and 100% [30,31]. The main advantage of RFR localization over wire localization is the decoupling of the radiology and surgery schedules; moreover, it avoids the risk of complications associated with an external wire component. Compared to RSL, RFR is a non-ionizing system and does not require extensive multidisciplinary coordination or regulatory compliance. Disadvantages of localization with the SAVI SCOUT device include its relatively large size (12 mm), especially for small subcentimetric lesions. The LOCalizer overcomes the size hurdle since it is smaller. Other limitations include the inability to reposition the reflector once deployed and the maximum lesion detection depth, as studies have reported problems in intraoperative detection of the reflector in women with large breasts and lesions located >6 cm from the overlying skin surface [30]. 

## 8. Intraoperative Ultrasound

Intraoperative ultrasound (IOUS) was first described by Schwartz et al. in 1988 and has gradually spread and evolved with other techniques due to growing experience and technological advances. A sterile-gowned ultrasound probe has to be available in the operating room. The procedure begins at the operating table before incision, once painting and draping procedures have been carried out. The surgeon locates the tumor by ultrasonography and measures its diameter and distance from surrounding hallmarks, such as skin surface, nipple–areolar complex (NAC) and fascia. The surgical approach is then planned in full liberty, and after incision the dissection is carried out by repeatedly reassessing the tumor’s position and the distance between the surgical plane and its margins. Once the excision has been completed, specimen ultrasound is performed at the operating table to assess margins, and additional shaving excisions can be acquired if necessary. This technique is highly effective, with identification rates close to 100% [31,32,33,34,35], and studies focusing on margin status have shown that IOUS guided surgeries yield less positive resection margins compared to WGL [32], with free-margin percentages ranging from 81% to 97% [33,34,35]. Free margin rates are enhanced by IOUS even in resection of palpable lesions [60]. A study by James et al. has instead shown no significant differences in margin status between IOUS and mammographic WGL in patients undergoing surgery for carcinoma in situ, although the authors still recommend performing IOUS as it is more cost-effective [61]. Compared to other techniques, IOUS yields several practical advantages: it does not increase patient presurgery psychological stress, as it is non-invasive compared to techniques involving breast compression or puncture; it grants full liberty to the surgeon in choosing the most convenient oncoplastic surgical approach; it does not aggravate organizational problems and coordination between several departments, as it takes place directly in the operating room and can be carried out completely by the surgeon himself [62]. To this regard, the learning curve of specialists not necessarily familiar with manipulating ultrasounds, such as surgeons, could potentially pose an issue, however a study by Krekel et al. suggests that performance of eight procedures is enough for the surgeon to acquire the expertise necessary to combine ultrasounds to palpation-guided surgery [63]. Drawbacks include technical problems resulting from combining ultrasound with surgery, such as air infiltration beneath the probe that can impede visualization, and refraction issues that can arise when scanning tissue that is irregular in shape [32]. The major, insurmountable issue of this technique is however represented by its inability to localize sonographically invisible tumors. To overcome this problem, some authors have described this technique in combination with hematoma-guided surgery after MRI- or stereotactically-guided biopsies, with mixed results [64].

## 9. Preoperative Localization with a Skin Tattoo

**Figure 4:**
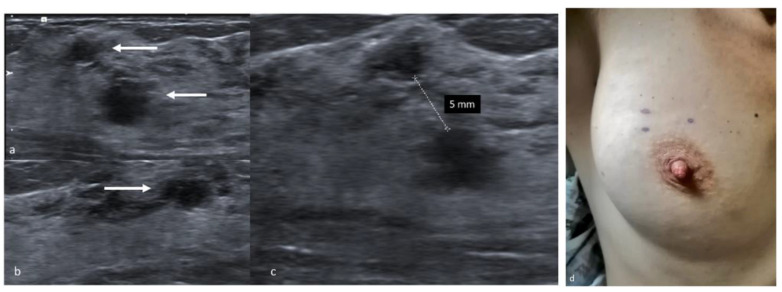
Preoperative skin tattoo. Transverse sonogram showing hypoechoic, round shaped multifocal masses with indistinct margins in the upper outer quadrant of the right breast (**a**,**b**, arrows). The distance between separate lesions is measured (**c**). The dermographic skin markers of the tumor’s projection on the skin surface (**d**).

Preoperative localization with a skin tattoo is a simple and safe technique amply utilized in our centre, as it is easily performed, extremely well tolerated by patients and effective in terms of successful excision and clear margin rates. This method can be carried out by acquiring either sonographic or mammographic images, depending on the type of lesion, but ultrasounds are employed whenever possible because the procedure is easier. In this case, patients lie in the supine position with their arms extended to mimic the position held during surgery. The tumor is located, and its distance from the skin surface is measured taking care not to apply pressure with the probe, so as to report accurately the depth of the tumor in relation to the skin surface [65]. The distance between the lesion, the nipple and the pectoralis major muscle is also measured, as is the distance between separate lesions in case of multifocal or multicentric disease [44,66]. Radiologists with experience in this technique visualize the tumors at their largest diameter to achieve the optimal correspondence between the lesion and the skin markers. The tumor’s projection on the skin surface is pinpointed with a dermographic skin marker and the drawing is covered to avoid accidental erasure (Figure 4). The whole procedure, performed by an experienced radiologist, takes 5–10 min and provides minimum patient discomfort. Limitations include poor results in case of sonographically invisible lesions, microcalcifications or biopsy markers, but are easily overcome by implementing this technique with a mammographic approach. Stereotactic-guided skin marking is also a non-invasive technique, albeit it provides a little more discomfort to the patient due to breast compression. Mammograms are acquired in double projection and measurements are performed on the images to determine the distance between the lesion and the nipple, the skin surface and the fascia.

**Figure 5:**
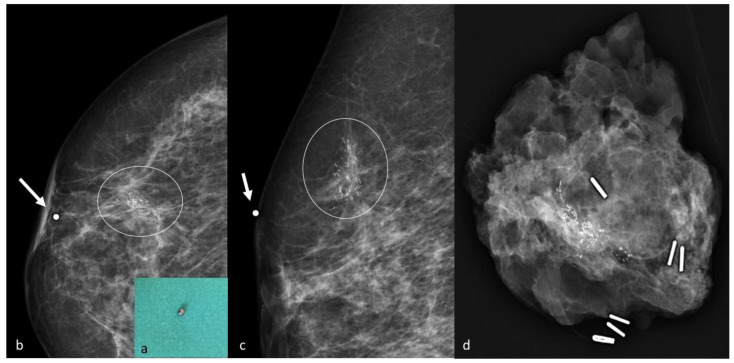
Lead marker positioning during mammographic technique. Metallic marker (**a**). Craniocaudal (**b**) and mediolateral oblique (**c**) views confirm the appropriate marker (arrow) placement on the microcalcifications’ (circle) projection on the skin surface. Specimen X-ray contains the microcalcifications (**d**).

**Figure 6:**
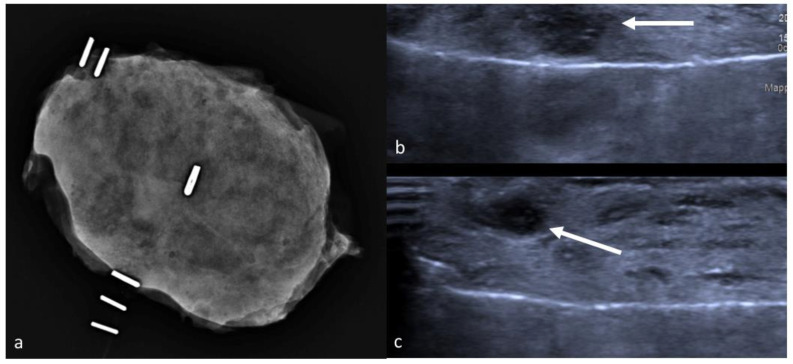
Radiograph of a dense, glandular specimen with scarcely recognizable nodules (**a**). Subsequent specimen ultrasound demonstrates successful removal of two masses (arrows) (**b**,**c**).

The radiologist then estimates the projection of the tumor on the skin surface and positions a lead marker in the corresponding spot. In case of bigger lesions, such as extensive microcalcifications, or multifocal disease, multiple lead markers can be employed to determine lesion margins. A second stereotactic pair of images is acquired to confirm the correct localization, and in case of inaccurate positioning, the lead markers can be repositioned more accurately and confirmed by a further mammogram [67] (Figure 5). At the end of the procedure the lead markers are removed, and the skin tattoo is drawn in their place. In the operating room, the mark is exposed and retraced with a specific marker resistant to antiseptic solutions, and painting and draping procedures are carried out carefully without wiping out the ink. Our centre strongly advocates pursuit of the maximum aesthetic result achievable with oncological safety, and because this localization technique employs only a temporary skin tattoo, the surgeon is granted total liberty in choice of incision and oncoplastic technique. The skin flap is dissected in the direction of the tattoo, then the incision is deepened and a lumpectomy is carried out taking into account tumor depth measured during the preoperative localization. In some cases, a non-palpable lesion becomes palpable after dissection of the skin flap, allowing the surgeon to easily complete the excision, however in most cases the excision has to be conducted by reassessing the original position of the skin mark from time to time. Once the excision is completed, metallic clips are placed on the orienting sutures in different numbers, so as to recognize margins in the specimen X-ray. The sample is then placed into a transparent plastic bag and sent to the Radiology Department, and mammograms are acquired in double projection. The tumor is usually visible as a radiopaque nodule, and its position inside the lumpectomy specimen is described as either well centered or close to one or more surgical margins, and reported to the operating surgeon. In dense, glandular specimens the nodule can be difficult to distinguish from the surrounding mammary parenchyma: in these cases, the exam can be completed with a specimen ultrasound [68] (Figure 6). If close margins are detected in either technique the surgeon can acquire further cavity shave margins on the affected border.

This technique is quick, easily performed by breast radiologists and extremely cost-effective. It does not require equipment that is not normally present in any breast surgery department, and is therefore feasible even with scarce resources. Limitations include accurate scheduling to time the procedure before surgery thus avoiding accidental mark erasure, and a certain degree of experience by the surgeon in reassessing the tumor’s position based on the skin mark during dissection. Reports on this technique are widely deficient in the literature, however a preliminary analysis of the data from our high-volume centre examining the outcome of 199 lumpectomies performed for non-palpable breast tumors between August and December 2019 identified a global success rate of 99.5% (198/199) and a clear margins rate of 95.9% (192/199). As these rates did not differ significantly from other localization techniques, this method appears safe and especially ideal in the case of limited resources or spending reviews.

## 10. Conclusions

Image-guided preoperative localization of breast lesions is a common procedure that has rapidly evolved throughout the last decades. Continuous technological developments and results from new clinical trials have provided growing insight and new possibilities for breast specialists to select upon various effective techniques. However, to date, no single perfect method exists. Therefore, the optimal approach should be tailored on each patient by taking into account preoperative disease characterization (both radiologic and histologic) and consulting all stakeholders, including surgeons, radiologists and pathologists.

## Figures and Tables

**Table 1:** Comparison of different localization techniques. Abbreviations: ROLL = radio-guided occult lesion localization; RSL = radioactive seed localization; Magseed = magnetic seed localization; IOUS = intraoperative ultrasound; Skin tattoo = preoperative localization with skin tattoo; OR = operating room; US = ultrasound; MRI = magnetic resonance imaging. * Success is defined as removal of target lesion. ** Authors’ experience.

Technique	Materials/Procedures	Advantages	Disadvantages	Success * Rate	Clear Margins Rate
Wire localization	Wire Preloaded needle introducer	Simple Cost-effective Different kinds of image-guidance	Wire migration Scheduling difficulties Limits surgical decisions	97.5% [14]	70.8–87.4% [15]
Carbon marking	Diluted charcoal powder	Simple Different kinds of image-guidance Cost-effective Cannot dislodge Scheduling flexibility	Carbon can distort or obscure lesion Unfit for large breasts Unfit for multifocal or extensive lesions	99% [16]	61–85% [17,18]
ROLL	Nuclear radiotracer Technetium 99 Gamma ray probe	Different kinds of image-guidance Does not limit surgeon	Scheduling difficulties Radiation Cost	95–99% [19]	92% [20,21]
RSL	Iodine 125 seed Preloaded needle introducer Gamma probe set for I-125	Scheduling flexibility Does not limit surgeon Different kinds of image-guidance	Radiation Not repositionable after deployment	100% [22,23]	73.5–96.7% [22,23]
Magseed	Paramagnetic seed Preloaded needle introducer	Scheduling flexibility No radiation Does not limit surgeon	Cost Not repositionable after deployment Non magnetizable surgical equipment MRI artifacts	99.86% [24,25,26,27]	88.75% [24]
Radiofrequency identification tags	Radiofrequency reflector Needle introducer Detector	Scheduling flexibility No radiation Does not limit surgeon	Cost Depth limit Not repositionable after deployment Interference with halogen lights in the OR	97–100% [28,29]	85–100% [28,29,30]
IOUS	Portable or OR-stationed US machine and sterile transducer cover	Scheduling flexibility No radiation Does not limit surgeon Non-invasive	Unemployable in US-invisible lesions Surgeon learning curve Interference with air during dissection	100% [31,32,33,34,35]	81–97% [32,34]
Skin tattoo	Dermographic marker Lead markers	Simple and safe Cost-effective Non-invasive Different kinds of image-guidance Does not limit surgeon	Scheduling difficulties Inability to depict marker	99.5% **	95.9% **
